# Risk of nosocomial respiratory syncytial virus infection and effectiveness of control measures to prevent transmission events: a systematic review

**DOI:** 10.1111/irv.12379

**Published:** 2016-03-24

**Authors:** Clare E. French, Bruce C. McKenzie, Caroline Coope, Subhadra Rajanaidu, Karthik Paranthaman, Richard Pebody, Jonathan S. Nguyen‐Van‐Tam, Noso‐RSV Study Group, Julian P. T. Higgins, Charles R. Beck

**Affiliations:** ^1^ School of Social and Community MedicineUniversity of BristolBristolUK; ^2^ NIHR Health Protection Research Unit in Evaluation of Interventions at University of BristolBristolUK; ^3^ University of NottinghamNottinghamUK; ^4^ Public Health EnglandLondonUK

**Keywords:** Infection control, nosocomial infections, palivizumab, personal protective equipment, respiratory syncytial virus

## Abstract

Respiratory syncytial virus (RSV) causes a significant public health burden, and outbreaks among vulnerable patients in hospital settings are of particular concern. We reviewed published and unpublished literature from hospital settings to assess: (i) nosocomial RSV transmission risk (attack rate) during outbreaks, (ii) effectiveness of infection control measures. We searched the following databases: MEDLINE, EMBASE, CINAHL, Cochrane Library, together with key websites, journals and grey literature, to end of 2012. Risk of bias was assessed using the Cochrane risk of bias tool or Newcastle–Ottawa scale. A narrative synthesis was conducted. Forty studies were included (19 addressing research question one, 21 addressing question two). RSV transmission risk varied by hospital setting; 6–56% (median: 28·5%) in neonatal/paediatric settings (*n *=* *14), 6–12% (median: 7%) in adult haematology and transplant units (*n *=* *3), and 30–32% in other adult settings (*n *=* *2). For question two, most studies (*n *=* *13) employed multi‐component interventions (e.g. cohort nursing, personal protective equipment (PPE), isolation), and these were largely reported to be effective in reducing nosocomial transmission. Four studies examined staff PPE; eye protection appeared more effective than gowns and masks. One study reported on RSV prophylaxis for patients (RSV‐Ig/palivizumab); there was no statistical evidence of effectiveness although the sample size was small. Overall, risk of bias for included studies tended to be high. We conclude that RSV transmission risk varies widely during hospital outbreaks. Although multi‐component control strategies appear broadly successful, further research is required to disaggregate the effectiveness of individual components including the potential role of palivizumab prophylaxis.

**What this paper adds**

Respiratory syncytial virus (RSV) transmission risk is substantial during outbreaks in hospital settings. Although multi‐component control strategies appear broadly successful in controlling nosocomial RSV transmission, there is a lack of high‐quality evidence and further research is required to identify the effectiveness of discrete measures including the role of palivizumab prophylaxis.

## Introduction

Respiratory syncytial virus (RSV) causes a significant public health burden; a systematic review and meta‐analysis estimated that globally the infection caused 33·8 million (95% confidence interval [CI] 19·3–46·2 million) new episodes of acute lower respiratory tract infections in children <5 years old in 2005.^1^ It is an important cause of severe respiratory disease in children, particularly those at high risk of acute lower respiratory tract infections.^2^, ^3^ RSV is also common in adults, especially the elderly and other high‐risk groups such as those who are immunocompromised.^4^, ^5^, ^6^ RSV outbreaks among vulnerable hospitalised patients are of particular concern as affected patients are more likely to experience longer hospital stays, with increased risk of morbidity and mortality.^4^, ^7^, ^8^ Numerous hospital outbreaks have been reported in multiple age groups and settings including neonatal intensive care, haematology, transplant and oncology units.^9^, ^10^, ^11^, ^12^

RSV infection does not lead to long‐term immunity.^13^ There is currently no specific treatment for RSV nor a licensed vaccine,^14^ so controlling transmission is crucial. RSV is transmitted via large nasopharyngeal secretion droplets from infected individuals.^15^ These droplets enter via the mucus membranes of the eyes, nose and mouth following close contact, or self‐inoculation after touching contaminated surfaces.^15^ Standard (routine) respiratory infection control procedures such as isolation of cases, high standards of hand hygiene, cohort nursing and use of personal protective equipment (PPE) have been reported as effective, to varying degrees, in the prevention and control of RSV outbreaks in nosocomial settings.^16^, ^17^, ^18^ In more recent years, immunoprophylaxis with the monoclonal antibody palivizumab has been used during hospital RSV outbreaks for patients at high risk of severe complications (e.g. preterm infants).^10^, ^11^, ^12^, ^19^, ^20^ To our knowledge, the effectiveness of RSV‐specific infection control measures in the hospital setting have not been subject to a high‐quality systematic review. A Cochrane review of physical interventions to prevent respiratory virus infections was published in 2011, but this was not specific to RSV or acute settings and did not seek to identify studies reporting on the effectiveness of palivizumab.^21^

We aimed to address the aforementioned gaps in the evidence base through a systematic review of the published and unpublished international literature. The specific research questions were as follows: (i) What is the risk of nosocomial RSV transmission where patients may have been potentially exposed to the infection (epidemiologically suspected or microbiologically confirmed) during an outbreak in a hospital ward or unit? and (ii) What is the effectiveness of infection prevention and control measures to minimise nosocomial transmission of RSV in the hospital setting?

## Methods

### Protocol registration and study conduct

The review protocol was registered with the PROSPERO International prospective register of systematic reviews, registration number: CRD42013003835.^22^ It was conducted following the general principles of the Cochrane Handbook for Systematic Reviews of Interventions,^23^ and is reported according to the requirements of the Preferred Reporting Items for Systematic Reviews and Meta‐Analyses (PRISMA).^24^

### Search strategy

We searched the following databases using MeSH and free‐text terms: MEDLINE, EMBASE, CINAHL and the Cochrane Library (CENTRAL). Bandolier and the Cochrane Library (CSDR, DARE, NHS HTA databases) were searched for evidence‐based reviews, and NHS Evidence (NHS Clinical Knowledge Summaries and the National Library of Guidelines) to identify guidelines containing relevant data. Two of the most relevant journals (Influenza and Other Respiratory Viruses and Eurosurveillance) were hand‐searched. Additional searches were conducted via Google, the Health Protection Agency website (now Public Health England [PHE]), the World Health Organization and the US Centers for Disease Control and Prevention. Experts in the field were consulted. Grey literature was sought via the Web of Science, NHS Evidence and OpenSIGLE. Reference lists of the most relevant records (~100) were searched, and Web of Science (Science Citation Index) and Google Scholar were used for citation tracking. Unpublished epidemiological data were sought from the PHE respiratory outbreaks database.

Our search strategy was designed to identify studies providing data addressing either or both research questions. The generic list of search terms is available in the protocol,^22^ and the full electronic search strategy for MEDLINE in Appendix 1. Searches were executed in February/March 2013 and included publications from the inception of each database to the end of 2012, in the English language. Searches were limited to humans. No restriction was placed on either the publication type (e.g. abstracts, unpublished works etc. were eligible) or study design. Review papers were not eligible for inclusion but were obtained for reference list searching.

### Study selection (inclusion and exclusion criteria)

Search records were imported into Endnote. After removal of duplicates, records were assessed for eligibility using a three‐stage sifting process sequentially reviewing titles, abstracts and full texts. Each record was independently assessed by two reviewers with the involvement of a third reviewer to resolve disagreements. To be eligible, studies had to address at least one of the two research questions. Only studies conducted in hospital settings were eligible. We were interested in clinically suspected RSV or bronchiolitis, or microbiologically confirmed RSV, epidemiologically suspected to be nosocomial in origin. We accepted any description of a nosocomial (rather than community) transmission, according to the authors’ own definition, as eligible. No restriction was placed on laboratory technique for identifying RSV.

To be eligible for inclusion for question one, studies had to (i) provide data on the risk of nosocomial RSV transmission to patients (attack rate), defined as follows: number of nosocomial RSV or bronchiolitis cases/number of patients potentially exposed to RSV in the ward or unit; and (ii) be conducted during an outbreak in a hospital ward or unit, defined as two or more cases of RSV infection linked epidemiologically in time and place or microbiologically confirmed. Studies providing data for a whole RSV season or routine surveillance data were not eligible. Research question two was defined as follows: Population: patients, staff or visitors at risk of RSV infection in the hospital setting; Intervention: RSV infection control measures; Comparator: infection control measures which differ from the intervention, or no intervention; and Outcome: nosocomial RSV transmission in the intervention versus comparator group. For question two, studies had to (i) state one or more RSV infection control interventions, (ii) utilise a comparator group and (iii) provide data on nosocomial RSV transmission in the intervention versus comparator groups, with no restriction placed on the type of data that were reported (e.g. risks or rates, risk or rate ratios, the ratio of RSV cases that were nosocomial). For question two, studies were not restricted to those conducted in the context of a specific outbreak (e.g. routine surveillance data comparing two RSV seasons were eligible). Studies which assessed the use of palivizumab to prevent RSV outbreaks were eligible for inclusion. Assessing the effectiveness of season‐long palivizumab prophylaxis for individual high‐risk patients or severity of RSV infection was beyond the scope of this systematic review.

### Data extraction

Two reviewers independently extracted data using a pre‐defined template. Disagreements were resolved by discussion or by a third reviewer. For research question one, we extracted the following: country and year of outbreak, hospital setting, study objective and nosocomial transmission risk (attack rate, number of nosocomial cases, number of patients at risk). For research question two, we extracted the following: country and year of outbreak, study design, hospital setting, infection control measures for intervention and comparator groups, and information on effectiveness of control measures.

### Risk of bias assessments

We assessed risk of bias, by domain, for all studies providing comparative data on the effectiveness of infection control interventions (i.e. addressing research question two). Experimental and prospective cohort studies were assessed using the Cochrane risk of bias tool,^23^ and retrospective cohort studies using the Newcastle–Ottawa scale.^25^ Abstracts, conference posters or proceedings were not assessed formally due to the limited information available.

### Data synthesis

A narrative approach was used to synthesise the extracted data and quality assessments according to the framework described by the Economic and Social Research Council and recommended by the University of York Centre for Reviews and Dissemination.^26^ Due to weak study designs and heterogeneity between studies, including the range of different control measures applied with most studies implementing multicomponent measures, it was not considered appropriate to carry out a meta‐analysis.

## Results

### Included studies

**Figure 1:**
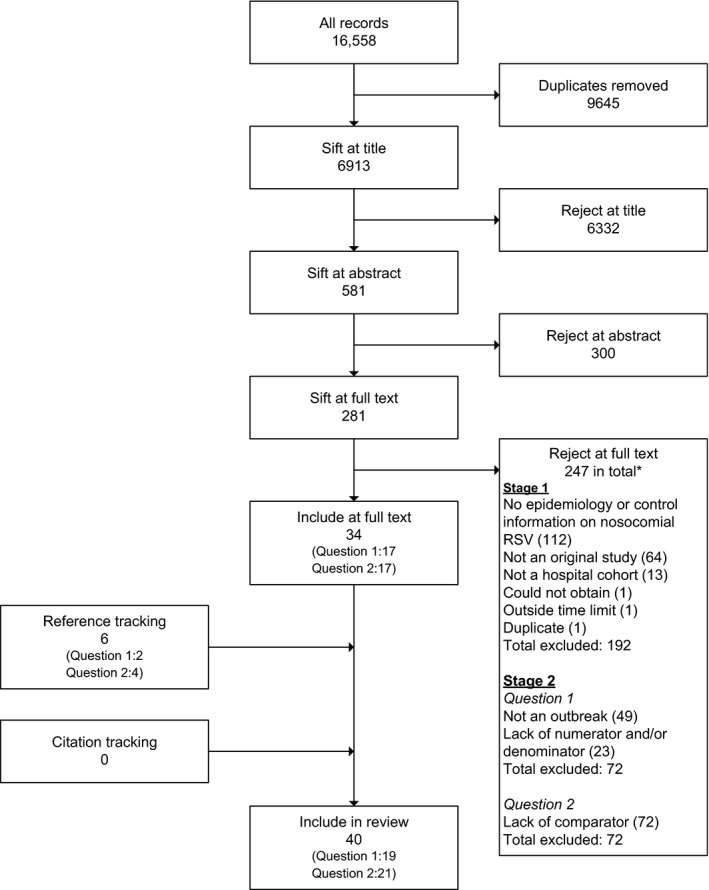
Study selection flow chart. *Note: As we used a single search strategy for the two research questions, a first sift of full‐text records was used to exclude records that were clearly not eligible for inclusion in the review as a whole. Each of the remaining studies (*n* = 89 [281–192]) was then independently assessed for eligibility for each of the two research questions.

The searches returned 16 558 records, 6913 after removal of duplicates, with an additional six studies obtained through reference list scanning. Forty studies were eligible for inclusion, 19 addressing research question one and 21 addressing research question two (none addressed both) (Figure 1). One outbreak recorded in the database held by the PHE Respiratory Diseases Department met the eligibility criteria for research question two.

### Risk of nosocomial RSV transmission

Table 1 summarises the 19 studies providing data on the risk of nosocomial RSV transmission. Eight were from Europe, six from the United States and five from elsewhere. Most (*n* = 14) were in neonatal/paediatric units (13 of which were neonatal units),^19^, ^20^, ^27^, ^28^, ^29^, ^30^, ^31^, ^32^, ^33^, ^34^, ^35^, ^36^, ^37^, ^38^ three in adult units for haematological cancers and/or bone marrow/stem cell transplant recipients (hereafter referred to as immunocompromised adults),^39^, ^40^, ^41^ and two in other adult units (a psychiatric ward and a continuing‐care ward for the elderly).^42^, ^43^ In all outbreaks, either all or the majority of diagnosed RSV cases were laboratory confirmed. The extent of case searching varied. The number of persons at risk ranged from 9 to 60 in neonatal/paediatric settings, 60–195 in adult units for immunocompromised adults and 25–27 in other adult units. RSV transmission risk varied by hospital setting: from 6% to 56% (median: 28·5%) in neonatal/paediatric settings, 6–12% (median: 7%) in units housing immunocompromised adults and 30–32% in other adult care settings. All studies utilised at least some type of infection control measures (either in place prior to the outbreak or implemented in response to it). The outbreak reported in the PHE database was in an adult haematology unit that included bone marrow transplant recipients. There were 20 persons at risk and the RSV transmission risk was 30%.

**Table 1:** Studies reporting on the nosocomial RSV transmission risk (research question one), ordered by hospital setting

Author, publication year	Country, year of outbreak	Hospital setting	Study objective^a^	Nosocomial cases/patients at risk (%)	Infection control measures^b^
Alan, 2012^28^	Turkey, 2012	Neonatal	To report on palivizumab use to control an RSV outbreak	1/16 (6·3)	Unclear; ‘strict contact measures’ for all patients given palivizumab; index cases cared for in separate isolation rooms
Boedy, 1988^29^	NC; assume USA, NS	Neonatal	Reports on containment, therapy and cost of a nosocomial RSV outbreak	8/35 (22·9)	Contact isolation, transfer of affected infants to separate unit to be cared for by infected healthcare workers, ribavirin treatment in 7 of 8 cases (for 3–5 days)
Dizdar, 2010^19^	Turkey, 2009	Neonatal	Reports an RSV outbreak in a NICU detected during a screening trial for RSV infection	15/50 (30·0)	Cohorting, gloves, gowns and masks, unit closed to elective admissions, visiting restrictions, screening of symptomatic staff, palivizumab prophylaxis
Halasa, 2005^30^	USA, 2002	Neonatal	To assess the medical and economic impact of a RSV outbreak in a NICU	9/56 (16·1)	Testing of all infants, isolation of infected infants, gowns and gloves, handwashing, palivizumab prophylaxis for all non‐infected infants, restriction of visiting policy (none <13 years), exclusion of staff and visitors with respiratory symptoms, NICU closed to non‐emergency admissions, infants who had been RSV‐positive were kept separated from non‐infected infants for 3 weeks, emergency admissions in room not occupied by those infected, RSV‐negative infants with respiratory symptoms isolated from other infants for 8 days
Kilani, 2002^31^	Saudi Arabia, 1999	Neonatal	To describe an RSV outbreak in a NICU and its successful control by infection control measures	8/20 (40·0)	Screening of all staff and infants, index cases isolated, evacuated rooms cleaned and fumigated, infant and staff cohorting, universal precautions with strict adherence to gowns/masks/gloves
Kokstein, 2001^32^	Czech Republic, 2000	Neonatal	Describes an RSV outbreak in a NICU	5/9 (55·6)	Barrier nursing, palivizumab for highest risk neonates
Kurz, 2008^33^	Austria, 2007	Neonatal	Reports on experience of using palivizumab and infection control measures to prevent RSV outbreaks in NICU	1/11 (9·1)	NICU closed to new admissions, masks, gloves and gowns used in care of all patients, visits restricted to mothers only (using gloves and masks); palivizumab administered to all patients
Meissner, 1984^34^	USA, 1982	Neonatal	Reports on a simultaneous outbreak of RSV and parainfluenza virus type 3 in a newborn nursery	9/34 (26·5)	Cohorting of infants and staff, ward closed to new admissions
Mintz, 1979^35^	USA, 1978	Neonatal	Reports on direct immunofluorescence to rapidly identify infants with RSV infection	7/17 (41·1)	RSV fluorescent antibody screening and viral cultures performed on all patients, patient isolation, strict handwashing, gowning and gloving procedures (masking was not required), cohort nursing, staff with upper respiratory symptoms were allowed to care for infected infants
Silva, 2012^20^	Brazil, 2010	Neonatal	To report on infection control measures and passive immunotherapy to control an RSV outbreak	10/18 (55·6)	Cohorting, masks, prophylaxis with palivizumab, immunoassay testing
Visser, 2008^36^	South Africa, 2006	Neonatal	To describe the molecular epidemiological investigation of an outbreak of RSV‐associated pneumonia	23/44 (52·3)	None specified
Salecedo, 2000^38^	Spain, 2000	Neonatal	Reports on the use of palivizumab prophylaxis to control an RSV outbreak	4/60 (6·6)	Palivizumab prophylaxis
White, 1990^27^	UK, 1990	Neonatal	To report on an RSV outbreak in a special care nursery	4/12 (33·3)	Isolation and cohort nursing, unit closed to admission for 2 days, new admissions separated from all existing admissions (infected or potentially infected), repeated screening of potentially infected babies, nurses with respiratory symptoms were designated to care for infected babies.
Krause, 1975^37^	USA, year not specified	Neonatal/paediatric	Reports on hospital staff and inpatient infants for carriage and acquisition of respiratory virus infections	4/15 (26·7)	None specified
Abdallah, 2003^39^	Australia, 2001	Adults – haematological cancers and/or bone marrow/stem cell transplant recipients	Describes the outcome of RSV‐infected inpatients during an outbreak	11/195 (5·6)	Isolation in a single room, handwashing, barrier protection including gowns, gloves and masks (no goggles), cohort nursing, screening for RSV if developed URTI symptoms, with isolation pending results, patients were advised to do the following: catching cough/sneeze, bin it and handwashing; thorough cleaning of patient room after discharge; exclusion of URTI‐symptomatic staff and visitors from patient care or visiting; staff education and written protocol on inpatient RSV management
Harrington, 1992^40^	USA, 1990	Adults – haematological cancers and/or bone marrow/stem cell transplant recipients	To describe an RSV outbreak in a bone marrow transplant centre	23/191 (12·0)	Usual universal precautions and additionally, masks for all persons entering room and for outpatients when indoors, RSV‐infected outpatients isolated when outpatient department, family and employees known to be infected excluded from hospital and outpatient department
Mangi, 1998^41^	UK, 1997	Adults – haematological cancers and/or bone marrow/stem cell transplant recipients	To report an outbreak of RSV infection in an adult leukaemia unit	4/60 (6·7)	Isolation, no children on ward, handwashing, staff screening
Huang, 2009^42^	Taiwan, 2005	Adults – other	To report on experience of containing an RSV outbreak in a psychiatric ward	8/25 (32·0)	Masks, gloves, gowns, handwashing, isolation cubicles, patient information and training, exclusion of staff with early onset symptoms, testing
Mandal, 1985^43^	UK, 1982–1983	Adults – other	Describes an outbreak of RSV infection in a continuing‐care elderly ward	8/27 (29·6)	None specified

RSV, respiratory syncytial virus; NICU, neonatal intensive care unit; URTI, upper respiratory tract infection.

a Objectives explicitly stated by the study author begin with ‘To…’.

b Most of these measures were applied in response to the outbreak (although some may have already been in place prior).

### Effectiveness of control measures to prevent transmission events

Table 2 summarises characteristics of the 21 studies addressing research question two. Four were from Europe, 15 from the United States and two from Canada. There were 13 experimental or prospective cohort studies^15^, ^16^, ^18^, ^44^, ^45^, ^46^, ^47^, ^48^, ^49^, ^50^, ^51^, ^52^, ^53^ and seven retrospective cohorts^54^, ^55^, ^56^, ^57^, ^58^, ^59^, ^60^ (one was an abstract only and there was not enough information to identify the study type).^61^ Most (*n* = 18) were conducted in neonatal/paediatric settings,^15^, ^16^, ^18^, ^44^, ^45^, ^46^, ^47^, ^48^, ^49^, ^50^, ^51^, ^52^, ^53^, ^55^, ^56^, ^57^, ^60^, ^61^ with three in units housing immunocompromised adults.^54^, ^58^, ^59^ All studies employed laboratory confirmation of RSV diagnoses.

**Table 2:** Studies assessing the effectiveness of nosocomial RSV infection prevention and control measures (research question two)

Author, publication year	Country setting, study year	Hospital setting	Study objective^a^	Study design
Agah, 1987^44^	USA, 1984–1985	Paediatric	To assess RSV infection rates in staff exposed to RSV, comparing those who wore goggles and masks with those who did not	Experimental
Gala, 1986^45^	USA, 1984	Neonatal/paediatric	To evaluate an eye–nose goggle in reducing nosocomial RSV infection in patients and staff	Experimental
Garcia, 1997^54^	USA, 1992–1994	Adults – haematological cancers and/or bone marrow/stem cell transplant recipients	To assess the effectiveness of a multifaceted infection control strategy in limiting the nosocomial RSV infection among patients	Retrospective cohort
Gardner, 1973^55^	UK, 1970–1972	Paediatric	To measure the extent and clinical importance of viral cross‐infection	Retrospective cohort
Hall, 1981^15^	USA, 1979	Neonatal/paediatric	To evaluate the efficacy of use of gowns and masks on the rate of nosocomial RSV in infants and staff	Experimental
Hall, 1978^51^	USA, 1976	Paediatric	To evaluate methods to control the spread of RSV infection on an infants ward during a community outbreak	Prospective cohort
Isaacs, 1991^16^	UK, 1986–1989	Neonatal/paediatric	To investigate whether cohorting infants and handwashing will reduce the incidence of nosocomial RSV	Prospective and retrospective cohort
Karanfil, 1999^56^	USA, 1989–1997	Paediatric	To report on implementation of control measures to prevent nosocomial RSV transmission	Retrospective cohort
Katz, 2009^57^	USA, 1990–2008	Neonatal	To compare nosocomial RSV infection rate in a NICU before and after RSV prophylaxis	Retrospective cohort
Krasinski, 1990^46^	USA, 1987–1988	Paediatric	To determine the efficacy of assignment to cohorts to reduce nosocomial RSV transmission	Prospective cohort
Langley, 1997^52^	Canada, 1992–1994	Paediatric	To determine nosocomial RSV transmission, outcomes and infection control practices	Prospective cohort
Lavergne, 2011^58^	Canada, 1999–2003	Adults – haematological cancers and/or bone marrow/stem cell transplant recipients	To evaluate impact of an enhanced infection control programme on incidence of nosocomial‐acquired RSV and its consequences	Retrospective cohort
Leclair, 1987^47^	USA, 1982‐1985	Neonatal/paediatric	To investigate the efficacy of a vigorous infection control effort in reducing nosocomial RSV transmission	Prospective cohort
Macartney, 2000^48^	USA, 1988–1996	Neonatal/paediatric	To determine the cost‐effectiveness and cost–benefit of an infection control programme to reduce nosocomial RSV transmission	Prospective cohort
Madge, 1992^18^	UK, 1989‐1992	Neonatal/paediatric	To define the most effective infection control procedure for the prevention of nosocomial infection on wards with limited isolation facilities	Prospective cohort
Page, 2007^61^	USA, 1996–2002	Paediatric	Reports on a comprehensive RSV isolation policy to prevent nosocomial RSV transmission	N/A (abstract only)
Raad, 1997^59^	USA, 1994–1996	Adults – haematological cancers and/or bone marrow/stem cell transplant recipients	Reports on a multifaceted control strategy to reduce nosocomial RSV transmission	Retrospective cohort
Hall, 1977^53^	USA, NS	Neonatal/paediatric	To identify shedding patterns of RSV, spread of RSV infection within families (in a community setting) and nosocomial spread of RSV	Prospective cohort
Murphy, 1981^50^	USA, 1979	Paediatric	To examine the effects of various control methods on the acquisition of symptomatic respiratory infections among medical staff caring for infants with respiratory disease	Prospective cohort
Simon, 2006^49^	Germany, 1999–2002	Paediatric	To assess the local epidemiology of nosocomial RSV infections and evaluate the global efficacy of a complex intervention programme	Prospective cohort
Snydman, 1988^60^	USA, 1984–1986	Neonatal	To investigate the impact of additional infection control methods for nosocomial RSV	Retrospective cohort

RSV, respiratory syncytial virus; NICU, neonatal intensive care unit; PICU, paediatric intensive care unit; LRTI, lower respiratory tract infection; URTI, upper respiratory tract infection; NS, not specified.

a Objectives explicitly stated by the study author begin with ‘To…’.

Studies on the effectiveness of interventions to prevent nosocomial RSV transmission to patients and staff are summarised in Tables 3 and 4, respectively. We found no eligible studies on interventions to prevent transmission to visitors. A range of different outcome measures were reported in eligible studies including the nosocomial transmission risk before and after the intervention, the rate of transmissions (e.g. per number of patient‐days at risk) in the intervention versus control group, and risk or rate ratios.

**Table 3:** Effectiveness of infection control measures in preventing nosocomial RSV transmission to patients, ordered by intervention type

Author, publication year	Intervention type	Intervention^a^	Control	Transmission risk in intervention group (patients)^b^	Transmission risk in control group (patients)^b^	Statistics (e.g. risk ratio, rate ratio, *P*‐value)
Garcia, 1997^54^	Multicomponent	Multifaceted infection control strategy including screening of symptomatic patients, isolation, cohorting, gloves and masks, screening and restriction of visitors, staff screening, staff training	Pre‐intervention – Ward A: contact precautions (use of disposable gloves and gowns when caring for RSV patients). Ward B: protected environment with laminar airflow rooms, reverse isolation and no visitors	1·4%	6·3%	Risk ratio in control versus intervention periods: 4·5 (95% CI: 1·5–13·5, *P* < 0·01)
Hall, 1978^51^	Multicomponent	Isolation or cohorting of symptomatic infants, handwashing, gowns (for staff attending infants with respiratory symptoms), staff cohorting, isolation of high‐risk infants, limitation of visitors	Data from a previous study in the same hospital used as a comparison group. During this previous period, infants were on a large open ward with symptomatic infants confined to their cubicles. Movement of staff and visitors between cubicles was not limited. Handwashing and gowns were supposed to be employed but were not monitored. Other infection control procedures were not routinely utilised	19%	45%	Risk in infants during intervention versus control period: *P* < 0·01
Isaacs, 1991^16^	Multicomponent	Admissions with suspected RSV placed in a separate screened‐off area, staff cohorting, handwashing including alcohol rub, parents/visitors instructed on handwashing told to keep older siblings with colds away from play areas, staff instructed on importance of handwashing, reinforced on ward rounds	‘Standard procedures’ which included gowns for nurses but no cohorting or educational programme to emphasise the importance of handwashing to staff and visitors	Period 2 (post‐intervention): 0·59%, Period 3 (post‐intervention): 1·1%	Period 1 (pre‐intervention): 4·2%	Authors state: ‘The difference between the first and second periods (*P* < 0·001) and between the first and third periods (*P* < 0·01) was significant, but not between the second and third periods (*P* = 0·5)’
Karanfil, 1999^56^	Multicomponent	Two‐stage control plan: Stage 1 begins when the first RSV case of the season is admitted to the centre – guidelines sent to all staff and samples sent for RSV culture for any children <2 years admitted with bronchiolitis or pneumonia. Pending laboratory results, the child is placed on paediatric droplet precautions (isolation and gloves for anyone entering the patient's room plus masks and gowns for close patient contact). Stage 2 begins when the 5th patient hospitalised from the community is identified – all children <2 years with any respiratory symptoms are placed on droplet precautions and tested for RSV	Gowns and gloves for patient contact. Private room not required	During the two seasons after implementation of the control programme: 7·2% (95% CI: 4·1%‐10·2%) of RSV cases were nosocomially transmitted	During the 2 seasons before the control programme: 16·5% (95% CI: 10·7–22·2%) of RSV cases were nosocomially transmitted	*P* = 0·002, uncorrected χ2. Authors also note ‘The control program's success also has been sustained during the 6 years it has been in place. A χ2 test for trend shows a continued decrease in the proportion of cases nosocomially acquired when the six RSV seasons after the intervention are compared with the average of the 2 years before the control program (*P* < 0·0001)’
Krasinski, 1990^46^	Multicomponent	Rapid RSV screening and assignment to a cohort at admission for all patients. Restricted visitation. Gowns used for patient contact when soiling was likely. Staff infection control memoranda/education. Gloves/masks were not used	No routine RSV screening on admission, category‐specific isolation practices	January–April 1987 (with screening): 1·23 cases per 1000 patient‐days, and September 1987–April 1988 (with screening): 0·46 per 1000 patient‐days	November–December 1986 (before screening programme): 7·17 cases per 1000 patient‐days	*P*‐value comparing January–April 1987 (intervention) with November–December 1986 (control): 0·026; and comparing September 1987–April 1988 (intervention) to November–December 1986 (control): 0·007
Langley, 1997^52^	Multicomponent	Study compares data for 9 different centres with different infection control measures. All centres isolated RSV‐positive patients in single rooms or cohorted them. Centre 1: gowns (for direct patient contact), gloves (for direct patient contact), masks (for direct patient contact); Centre 2: gowns (for anyone entering room), gloves (for direct patient contact), masks (if aerosolised ribavirin being administered); Centre 3: gowns (for direct patient contact), no gloves, masks (for direct patient contact); Centre 4: gowns (for direct patient contact), no gloves, masks (if aerosolised ribavirin being administered); Centre 5: gowns (for direct patient contact), gloves (for direct patient contact), masks (for direct patient contact); Centre 6: gowns (for direct patient contact), gloves (for direct patient contact), masks (for anyone entering room); Centre 7: gowns (for anyone entering room), gloves (for direct patient contact), masks (if aerosolised ribavirin being administered); Centre 8: gowns (for direct patient contact), gloves (for direct patient contact), masks (if aerosolised ribavirin being administered); Centre 9: gowns (for anyone entering room), gloves (for direct patient contact), masks (for anyone entering room, only if aerosolised ribavirin being administered)	Note: There is no single control group. Please see details of the various intervention groups (previous box)	Nosocomial ratio (i.e. number of nosocomial RSV cases/all RSV cases): Centre 1: 3·8; Centre 2: 8·8; Centre 3: 5·5; Centre 4: 5·0; Centre 5: 3·2; Centre 6: 2·8; Centre 7: 9·0; Centre 8: 7·8; Centre 13·0	Note: There is no single control group.	Authors state: ‘No isolation policy was associated with decreased nosocomial ratio, although gowning for any entry to the patient's room was associated with increased risk of RSV transmission (incidence rate ratio: 2·81; 95% confidence interval: 1·65,4·77; *P* < .000) in the multivariate Poisson’
Macartney, 2000^48^	Multicomponent	Early recognition of patients with respiratory symptoms (high index of suspicion, laboratory testing), patient isolation where possible (otherwise patients were cohorted), staff cohorting, contact precautions for all patients with symptoms of viral respiratory tract infection (handwashing, gowns and gloves for staff), visitation restrictions, staff education and compliance monitoring	Methods used for prevention of RSV varied among patient care units. Screening of patients for RSV infection occurred; however, the use of barrier methods for isolation and cohorting of patients and nursing staff was inconsistent	0·73 cases per 1000 hospital days at risk	0·98 cases per 1000 hospital days at risk	Mantel–Haenszel relative risk in post‐intervention versus pre‐intervention period, stratified by intensity of exposure: 0·61 (95% CI: 0·53–0·69)
Madge, 1992^18^	Multicomponent	All children screened within 18 hours of admission during the three RSV seasons. Cohort nursing and/or gloves and gowns (for all contacts with patients)	All children screened within 18 hours of admission during the three RSV seasons. No special precautions (handwashing after all contacts and gowns/gloves for contact with bodily fluids)	Gowns/gloves (winter 1/2): 28%, Cohort nursing (winter 1/2): 19%, Cohort nursing/gowns/gloves (winter 1/2): 3%	26%	Authors state ‘Nosocomial transmission rate was significantly reduced by the combination of cohort nursing and wearing of gowns/gloves for all contacts of RSV‐infected children (*P* = 0·0022). Neither intervention alone resulted in a significant reduction’
Page, 2007^61^	Multicomponent	RSV isolation policy (introduced in 1996), followed by a review of the policy several years later with education emphasised, the initiation of a comprehensive hand‐hygiene programme	Not specified, assume standard procedures	After implementation of new RSV isolation policy: 4 cases in the first 4 years, 6 cases in 2000‐2001, 3 cases in 2001‐2002. After review of policy: 0 cases in the subsequent 4 RSV seasons	Seven cases in winter of 1995	Not reported
Raad, 1997^59^	Multicomponent	Multifaceted infection control strategy, including masking and droplet precautions (not standard practice), isolation, strict adherence to measures such as handwashing, gloving and gowning by anyone entering patient's rooms, masks for close contact with patients, screening symptomatic patients, visitor screening and restrictions, staff education	Standard procedures including handwashing, gloving and gowning. Droplet isolation precautions, such as masking were not recommended	Period 2 (1996, second year post‐intervention): incidence 0·2 cases per 1000 patient‐days	Period 1 (1994, pre‐intervention): incidence of 1·4 cases per 1000 patient‐days	Not reported
Hall, 1977^53^	Multicomponent	Following a move to a new hospital – isolation or cohorting of children with respiratory disease, gowns, strict handwashing, children not allowed to visit wards, objects (e.g. sheets, trays) considered as contaminated	Infants with respiratory illness confined to cribs	‘About 10%’	32%	Not reported
Simon, 2006^49^	Multicomponent	Multicomponent strategy including staff education, increased RSV vigilance, isolation or cohorting of infected patients, strictly enforced contact precautions (hand disinfection, gowns, masks and gloves, disinfection of non‐critical nursing items), and daily disinfection of hand contact surfaces in the isolation room. Compliance of healthcare workers and parents was routinely monitored	Not specified, assume standard procedures	2000–2001 (post‐intervention): 14·6% (*n* = 13); and 2001–2002 (post‐intervention): 3·6% (*n* = 2)	1999–2000 (pre‐intervention): 17·4% (*n* = 24)	*P* = 0·039 comparing the first and the last season
Snydman, 1988^60^	Multicomponent	Active surveillance, admission and transfer policy guidelines, patient isolation or cohorting, nursing staff cohorting, respiratory precautions on suspicion of respiratory illness, gowns, gloves and masks on contact, winter visiting policy (screening of visitors and visitation limitations), construction of segregate areas	Previous infection control policy (CDC guidelines were in use; private room and gown on contact)	1984–1985 (post‐intervention): 0 cases in 668 patient‐days at risk, and in 1985–1986 (post‐intervention): 0 cases in 1020 patient‐days at risk	1983–1984 (pre‐intervention): 7 cases in 875 patient‐days at risk	Transmission rate 1984–1986 (post‐intervention) versus 1983–1984 (pre‐intervention): *P* = 0·0016
Gala, 1986^45^	PPE	Eye–nose goggle worn by all staff when entering the room of any infant with respiratory symptoms	Standard procedures (handwashing, isolation and cohorting)	5·9%	42·9%	χ2: *P* = 0·04
Hall, 1981^15^	PPE	Gowns and masks with a change of gowns between infant contacts	Standard procedures (handwashing, isolation or cohorting of infected infants, staff cohorting, restricting young visitors, restricting patient contacts)	32·0%	40·7%	Authors state: ‘The rate of nosocomial infection occurring in the first period is not significantly different from that of the second period’ (no statistics provided)
Leclair, 1987^47^	PPE	Gloves and gowns, with staff compliance monitoring, for direct contact with any child with suspected or known RSV infection (compliance = 81%)	No monitoring of staff compliance with gowns and gloves for direct contact with any child with suspected or known RSV infection (compliance = 38·5%)	Incidence per 1000 patient‐days: 3·1	Incidence per 1000 patient‐days: 6·4	Relative risk in control versus intervention periods (adjusted for intensity of exposure to nosocomial RSV): 2·9 (95% CI: 1·5‐5·7)
Katz, 2009^57^	RSV prophylaxis	RSV prophylaxis (RSV‐Ig or palivizumab) for high‐risk infants in addition to standard procedures	Standard infection control procedures including use of single rooms or cohorting of infected infants and droplet/contact isolation	Period 2 (post‐RSV prophylaxis with RSV‐Ig): 3·1 per 10 000 patient‐days, Period 3 (post‐RSV prophylaxis with palivizumab): 0·63 per 10 000 patient‐days	Period 1 (pre‐intervention): 2·1 per 10 000 patient‐days	Rate ratio period 1 versus period 2: 0·67 (95% CI: 0·03‐14·0, *P* = 0·76); and period 1 versus period 3: 3·3 (95% CI: 0·16‐68, *P* = 0·37)
Lavergne, 2011^58^	Isolation strategy	Enhanced seasonal infection control programme – same as the ‘targeted infection control program’ (applied to the control group) but *all* patients hospitalised during the RSV season were isolated until discharge	Standard ‘targeted infection control program’ (isolation was applied only to patients with severe neutropenia (<500/mm^3^) or presenting symptoms of upper and/or lower respiratory tract infection). Infection control measures comprised: private rooms with filtered air positive pressure ventilation, mandatory handwashing, screening all people entering ward for respiratory symptoms, visitation restrictions, patients forbidden to leave rooms except for special examinations, mandatory masks, gowns and gloves for all, RSV patients moved to negative pressure rooms for, for example, ribavirin treatments, rapid diagnosis of infection	3·9 cases per 1000 admissions	42·8 cases per 1000 admissions	Relative risk in intervention versus control period: 0·09 (95% CI: 0·02–0·38)
Gardner, 1973^55^	Isolation strategy (ward design)	Wards composed almost entirely of individual cubicles	Open wards with some cubicles	Cross‐infection rate (number of cross‐infections × 10^6^/((number at risk × mean stay) × (number infected × their mean stay)): 4·2	Cross‐infection rate: 7·1	Not reported. Authors state that the numbers of cross‐infections were too small to make statistical comparisons.

RSV, respiratory syncytial virus; NICU, neonatal intensive care unit; CDC, United States Centers for Disease Control and Prevention.

a Note that interventions were generally applied *in addition* to the ‘standard precautions’ used for the control group.

b Or other measure of nosocomial RSV transmission as specified (if transmission risk not reported).

**Table 4:** Effectiveness of personal protective equipment in preventing nosocomial RSV transmission to staff

Author, publication year	Intervention type	Intervention^a^	Control	Transmission risk in intervention group (staff)^b^	Transmission risk in control group (staff)^b^	Statistics (e.g. risk ratio, rate ratio, *P*‐value)
Agah, 1987^44^	PPE	Goggles and masks supplemented routine isolation procedures. Gowns were used if soiling was likely	Routine isolation procedures but no goggles or masks. Gowns were used if soiling was likely	5% (‘RSV illness rate’)	61% (‘RSV illness rate’)	*P* < 0·01
Gala, 1986^45^, ^c^	PPE	Eye–nose goggle worn by all staff when entering the room of any infant with respiratory symptoms	Standard procedures (handwashing, isolation and cohorting)	5%	34%	*P* = 0·003
Hall, 1981^15^, ^c^	PPE	Gowns and masks with a change of gowns between infant contacts	Standard procedures (handwashing, isolation or cohorting of infected infants, staff cohorting, restricting young visitors, restricting patient contacts)	33·0%	42·3%	Authors state: ‘The rate of infection occurring in the first period compared with the second period is not statistically different’ (no statistics provided)
Murphy, 1981^50^	PPE	Gowns and masks, in addition to handwashing	Handwashing only	17·9% (5/28)	13·3% (4/30)	≥0·2

RSV, respiratory syncytial virus; PPE, personal protective equipment.

a Note that interventions were applied *in addition* to the ‘standard precautions’ used for the control group.

b Or other measure of nosocomial RSV transmission as specified.

c Note that these two studies also appear in Table 3.

#### Risk of bias assessments

Cochrane risk of bias assessments were carried out for the 13 experimental or prospective cohort studies (Table 5). For domains relating to selection and performance bias (random sequence generation, allocation concealment, blinding of participants and personnel), the risk of bias was deemed high for all but one study in which the risk for the first two domains was unclear^50^. No studies reported blinding of participants and personnel (that would largely not have been possible due to the nature of the interventions). Detection bias (blinding of outcome assessors) was considered low risk for all studies because RSV cases were laboratory confirmed. None of the studies sufficiently adjusted for potential confounding. An additional potential bias is the ascertainment of community‐acquired rather than nosocomial RSV cases. Eleven of the 12 studies investigating the risk of RSV transmission to patients provided a clear definition of a nosocomial case, with most (*n* = 8) defining this as a case occurring at least 5 days after hospital admission (some used a higher cut‐off).^15^, ^18^, ^45^, ^46^, ^47^, ^48^, ^49^, ^51^ In studies assessing the risk of transmission to hospital staff, no such case definition would be possible. The risk of attrition bias was unclear in most studies, with only two studies^44^, ^50^ providing relevant information. The risk of reporting bias was unclear for all studies.

**Table 5:** Cochrane risk of bias assessments for experimental and prospective cohort studies

	Random sequence generation	Allocation concealment (selection bias)	Blinding of participants and personnel (performance bias)	Blinding of outcome assessment (detection bias)	Incomplete outcome data (attrition bias)	Selective reporting (reporting bias)	Other sources of bias 1 (other bias – case definition)	Other sources of bias 2 (other bias – confounding)
Agah, 1987^44^	−	−	−	−	−	?	−	−
Gala, 1986^45^, ^a^	−	−	−	+	?	?	+	−
Hall, 1981^15^, ^a^	−	−	−	+	?	?	+	−
Hall, 1978^51^	−	−	−	+	?	?	+	−
Isaacs, 1991^16^	−	−	−	+	?	?	?	−
Krasinski, 1990^46^	−	−	−	+	?	?	+	−
Langley, 1997^52^	−	−	−	+	?	?	+	−
Leclair, 1987^47^	−	−	−	+	?	?	+	−
Macartney, 2000^48^	−	−	−	+	?	?	+	−
Madge, 1992^18^	−	−	−	+	?	?	+	−
Hall, 1977^53^	−	−	−	+	?	?	?	−
Murphy, 1981^50^	?	?	−	+	+	?	−	−
Simon, 2006^49^	−	−	−	+	?	?	+	−

Key: −: high risk of bias; +: low risk of bias; ?: unclear risk of bias.

a The studies by Gala *et al*. (1981) and Hall *et al*. (1981) both investigated RSV transmission to staff as well as patients. A ‘low’ rating has been assigned for the ‘other bias – case definition’ domain based on the nosocomial case definition used for RSV cases occurring among *patients*, but it should be noted that no such definition was applied to RSV cases occurring among staff.

Of the seven retrospective cohort studies assessed on the Newcastle–Ottawa scale^25^ (Table 6), most scored highly on selection of the study groups with six studies^54^, ^55^, ^56^, ^57^, ^58^, ^60^ awarded three or more stars for this domain. However, all studies received zero stars for ‘comparability’ as none adjusted for confounders or utilised a study design that matched individuals in the intervention and comparator groups. Although all but one study provided a clear definition of a nosocomial RSV case,^59^ ascertainment of the outcome was poor overall (generally due to insufficient follow‐up or inadequate reporting of this); six studies were awarded one star only for this domain^54^, ^55^, ^56^, ^57^, ^58^, ^60^ and one awarded zero stars.^59^

**Table 6:** Newcastle–Ottawa ratings for retrospective cohort studies

	Selection stars	Comparability stars	Outcome stars
Garcia, 1997^54^	* * *		*
Gardner, 1973^55^	* * * *		*
Karanfil, 1999^56^	* * *		*
Katz, 2009^57^	* * *		*
Lavergne, 2011^58^	* * *		*
Raad, 1997^59^	* *		
Snydman, 1988^60^	* * *		*

Note: A maximum of four stars can be awarded for ‘Selection’, two stars for ‘Comparability’ and three stars for ‘Outcome’. Where a box is blank, this is because zero stars were awarded.

#### Multicomponent interventions (Table 3)

Most studies (*n* = 13) employed multicomponent infection control strategies (two or more measures combined).^16^, ^18^, ^46^, ^48^, ^49^, ^51^, ^52^, ^53^, ^54^, ^56^, ^59^, ^60^, ^61^ These comprised a wide range of measures including the following: prompt RSV case‐finding among symptomatic patients; screening all patients on admission; screening staff and/or visitors; isolation policies and/or staff/patient cohorting; restriction of visitors (e.g. no young children); and staff training and/or compliance monitoring. Most studies made some use of personal protective equipment (PPE) (e.g. gowns, gloves, masks, goggles). Studies of multicomponent control measures essentially used ‘standard infection control precautions’, that is usual practice (either explicitly stated or assumed, see Table 3), as the comparator group. For example, data for previous RSV seasons prior to the introduction of the intervention were frequently utilised. Nine of the 13 studies presented evidence that nosocomial infections were significantly lower when a multicomponent intervention was implemented and provided supporting statistical data (e.g. *P*‐value or risk ratio with confidence intervals).^16^, ^18^, ^46^, ^48^, ^49^, ^51^, ^54^, ^56^, ^60^ Three studies reported data that were suggestive of a beneficial effect but did not present any supporting statistics (such as a *P*‐value).^53^, ^59^, ^61^ Relative risk reductions in transmission were variable but tended to be quite substantial and were in excess of 50% for the majority of studies, where calculable. One study using a multicomponent intervention also provided information on transmission to staff, although the risk was actually somewhat higher during the intervention than control period (56% versus 42%, no *P*‐value presented).^51^ However, Langley *et al*. 52 compared data for nine different hospitals using different combinations of intervention measures (all included isolation or cohorting) and concluded that RSV transmission to patients was not reduced by any type of isolation policy used, and there was no beneficial effect of a gloving or masking policy.

#### Staff personal protective equipment (Tables 3 and 4)

Five studies examined the use of staff PPE in addition to standard precautions, all conducted in neonatal/paediatric settings.^15^, ^44^, ^45^, ^47^, ^50^ Two examined the effect on RSV transmission to both staff and patients,^15^, ^45^ two examined the risk of transmission to staff only,^44^, ^50^ and the remaining study looked only at transmission to patients.^47^ Of the three studies providing data on patients (Table 3),^15^, ^45^, ^47^ two reported PPE to be effective. Gala *et al*. 45 implemented an eye–nose goggle for all staff and reported a transmission risk of 43% in the control and 6% in the intervention periods (*χ* ^2^: *P* = 0·04). Leclair *et al*. 47 examined the effect of monitoring staff compliance with PPE where it was hospital policy for staff to wear gloves and gowns when in direct contact with RSV patients. This study reported a lower risk of nosocomial transmission during the period in which intensive staff compliance monitoring was implemented compared to the pre‐intervention period (relative risk adjusted for intensity of exposure: 2·9 [95% CI: 1·5–5·7]). However, authors of the third study reported that they found no significant difference in the rate of nosocomial RSV transmission to patients when gowns and masks were used (32%) compared with standard procedures alone (41%) (no *P*‐value provided).^15^ Of the four studies examining the effectiveness of measures to prevent RSV transmission to staff (Table 4),^15^, ^44^, ^45^, ^50^ two found evidence of effectiveness, both of which utilised goggles (one used goggles and masks^44^ and the other an eye–nose goggle^45^). In the two studies reporting no statistically significant benefit, neither used goggles (just gowns and masks), although it should be noted that in both these studies the risk of transmission (based on the point estimates) was still lower in the intervention than the comparator groups.^15^, ^50^

#### RSV prophylaxis (Table 3)

Only one eligible study, in a neonatal unit, reported on post‐admission RSV prophylaxis for patients.^57^ Standard infection control procedures in the unit involved placing infected infants in single rooms or cohorting them and using droplet/contact isolation measures. The authors reported no significant difference in nosocomial RSV infection rates during the RSV seasons in which RSV prophylaxis (RSV‐Ig or palivizumab) was given monthly to all high‐risk infants in the unit in addition to standard infection control procedures: rate ratio for period 1 (no prophylaxis) versus period 2 (RSV‐Ig): 0·67 (95% CI: 0·03–14, *P* = 0·76); and for period 1 (no prophylaxis) versus period 3 (palivizumab): 3·3 (95% CI: 0·16–68, *P* = 0·37). However, the point estimates indicated a potential beneficial effect of palivizumab, and it should be noted that the power to detect a statistically significant difference was likely low due to the very small number of cases.^57^

#### Other interventions (Table 3)

One study compared the RSV transmission risk in wards comprising mainly of individual cubicles with the risk in open wards combined with a smaller number of cubicles. Although the numbers of nosocomial infections were too small to make statistical comparisons, the rate of nosocomial RSV infections was somewhat lower in wards composed largely of individual cubicles (7·1 versus 4·2 infections per million susceptible days per infective day).^55^ Meanwhile, in a haematology–oncology ward, isolating all patients hospitalised during an RSV season resulted in statistically significantly lower RSV transmission than the previous policy of only isolating patients with severe neutropenia or symptoms of upper and/or lower respiratory tract infection (relative risk in intervention versus control period: 0·09 [95% CI: 0·02–0·38]).^58^

## Discussion

### Key findings

To our knowledge, this is the first systematic review of nosocomial RSV transmission risk and the effectiveness of infection control measures to prevent transmission in acute care settings. Nosocomial RSV transmission risk is substantial during outbreaks in hospital settings, with notable variations across outbreaks and settings. Most studies in this review were conducted in neonatal/paediatric settings where the median transmission risk was 28·5%. While all studies utilised at least some infection control measures, the identified transmission risks are concerning and highlight a serious challenge. This review highlights the lack of high‐quality studies describing the effectiveness of infection control measures to prevent nosocomial RSV.

The majority of studies implemented multicomponent interventions, as appropriate for infection control in hospital settings and in accordance with, for example, UK guidelines.^62^ The evidence presented here broadly supports the use of multicomponent measures which tended to achieve relative reductions in transmission risk of over 50%. However, within this context, it was not possible to assess the effectiveness of individual components of control measures, either within individual studies or at the review level. Some components may be more effective than others, and identifying these could result in more efficient use of resources and further reductions in transmission risk. Furthermore, the potential harms of interventions, particularly those without measurable benefit, should not be overlooked.

Personal protective equipment worn by staff may potentially prevent transmission from patients to staff and *vice versa*. Two studies in which staff eye protection was used (eye–nose goggle^45^ or goggles plus masks^44^) found this to be effective in preventing transmission to staff (the first also reported a reduction in transmission to patients, but this was not investigated in the second study). This finding is consistent with RSV transmission generally occurring through the eye or nose.^63^ Evidence for the effectiveness of gowns and masks was lacking in two studies^15^, ^50^ although a further study did find high (versus lower) compliance with gloves and gowns to be effective at reducing nosocomial RSV transmission.^47^ In relation to the transmission of RSV to staff, there were four eligible studies, all of which investigated PPE as the intervention of interest. Of course, PPE is not the only potentially effective precaution; for example, staff/patient cohorting may also prevent transmission. Meanwhile, strict isolation precautions appeared to be effective in the two studies investigating this specifically,^55^, ^58^ but the resource implications of such policies (e.g. isolating all patients hospitalised during the RSV season in a given unit)^58^ may make them impractical to implement in many settings, especially in low‐ and middle‐income countries.

Although palivizumab is considered to be effective in preventing RSV‐related hospitalisation in high‐risk children (outside the scope of this review),^64^, ^65^, ^66^ we uncovered limited evidence regarding its use in hospitalised patients to prevent nosocomial RSV transmission. Our literature search returned 12 studies; however, only one of these met the review eligibility criteria for research question two.^57^ Of the 11 other studies (all of which lacked a comparator group), all but one were conducted in NICUs. Eight reported no further RSV cases following palivizumab prophylaxis,^10^, ^11^, ^28^, ^32^, ^33^, ^38^, ^67^, ^68^ and one reported no further cases after day 14 of the outbreak having instigated control measures on day nine.^30^ Two reported the occurrence of two further cases after palivizumab administration.^19^, ^69^ Meanwhile, Silva *et al*. 20 documented the occurrence of 10 RSV cases following prophylaxis administration to all patients, although the authors noted these infants may have already been in the RSV incubation period when palivizumab was administered. These additional data underscore the need for high‐quality studies in hospital settings to generate robust evidence to support clinicians and public health policy, particularly bearing in mind the high cost of palivizumab.^70^

### *Limitations*

RSV is a significant problem across low‐ and middle‐income countries.^1^ However, the majority of evidence on the risk of RSV transmission, and all evidence on interventions to interrupt transmission in this review came from the United States and Europe. Extrapolation of our findings to low‐ and middle‐income countries where resources are lacking may be difficult.

To calculate the nosocomial RSV attack rate, the denominator was the number of persons at risk of infection as few studies provided the person‐time at risk. Although this is a crude denominator, it allowed for comparisons across studies. The definition of a nosocomial transmission event varied between studies and we accepted any description of a nosocomial event, as defined by the author, as eligible. This may have resulted in some misclassification between nosocomial and community‐acquired RSV. Additionally, patients infected with RSV in hospital who did not develop symptoms until after discharge, were likely not identified by the studies, especially if symptoms were mild and they did not require re‐admittance to hospital (most studies did not report following up patients after discharge). Also, we cannot discount the potential for under‐reporting of nosocomial RSV outbreaks leading to reduced external validity of our findings.

We did not identify any randomised controlled trials on the effectiveness of RSV infection control measures. Observational studies are subject to inherent biases and furthermore, assessing the risk of bias in non‐randomised studies is difficult in itself.^23^ On the whole, available studies were assessed as having a relatively high risk of bias. A number of the studies utilised comparator data from a different time period (such as prior RSV seasons) and thus are subject to confounding due to differences between the population groups and levels of exposure to RSV. Studies typically did not clearly report the population characteristics of the two groups or control any potential differences, thus making comparisons difficult and potentially subject to bias. Few studies monitored compliance with infection control measures, which has been reported to frequently be suboptimal.^71^ High levels of compliance may be necessary for certain infection control measures to be effective. Finally, it should be noted that a number of the studies were poorly reported with a lack of clarity, for example, with regard to the study population and type/timing of interventions. Reporting of future studies in line with the ORION (Outbreak Reports and Intervention Studies Of Nosocomial infection) statement would improve their usefulness.^72^

## Conclusion

RSV transmission risk varies widely during hospital outbreaks. Although there is a lack of high‐quality evidence, multicomponent control strategies appear broadly successful, while PPE interventions using eye protection appear more effective than those using gowns and masks. Further research is required, especially in low‐ and middle‐income countries, to identify the most effective and cost‐effective individual control measures including the potential role of palivizumab prophylaxis during nosocomial outbreaks.

## Authors’ contributions

Authorship list: Clare E. French (CEF), Bruce C. McKenzie (BCM), Caroline Coope (CC), Subhadra Rajanaidu (SR), Karthik Paranthaman (KP), Richard Pebody (RP), Jonathan S. Nguyen‐Van‐Tam (JSN‐V‐T), Noso‐RSV Study Group, Julian PT Higgins (JPTH), Charles R. Beck (CRB). BCM, SR, KP, RP and CRB were involved in the review concept and design. BCM, SR and CRB designed and executed the searches. CEF, BCM, CC, SR, KP, the Noso‐RSV study group, JPTH and CRB contributed to study selection, data extraction and risk of bias assessments. CEF, CC, JPTH and CRB were involved in data analysis. CEF drafted the manuscript. All authors contributed to the interpretation of the data and writing of the final manuscript and approved it for submission.

## Disclosure and competing interest statement

CEF, BCM, CC, SR, KP, RP, JPTH: none to declare. JSN‐V‐T and CRB are respectively Editor‐in‐Chief and Associate Editor for Influenza and Other Respiratory Viruses; however they played no role whatsoever in the editorial process for this paper, including decisions to send the manuscript for independent peer‐review or about final acceptance of a revised version. All of the above functions were handled alone by Dr Alan Hampson.

## Appendix 1 MEDLINE search strategy

1. exp Hospitals/
2. exp Hospitalization/
3. exp Hospital Units/
4. exp Intensive Care Units/
5. exp Intensive Care Units, Neonatal/
6. exp Intensive Care Units, Pediatric/
7. exp Respiratory Care Units/
8. hospital*.mp.
9. ward.mp.
10. unit.mp.
11. icu.mp.
12. nicu.mp.
13. hdu.mp.
14. picu.mp.
15. 1 or 2 or 3 or 4 or 5 or 6 or 7 or 8 or 9 or 10 or 11 or 12 or 13 or 14
16. exp Infection Control/
17. exp Communicable Disease Control/
18. exp Infection Control Practitioners/
19. exp Quarantine/
20. exp Patient Isolation/
21. exp Patient Isolators/
22. exp Hygiene/
23. exp Anti‐Infective Agents, Local/
24. exp Disinfectants/
25. exp Protective Clothing/
26. exp Protective Devices/
27. exp Respiratory Protective Devices/
28. exp Gloves, Protective/
29. exp Masks/
30. exp Eye Protective Devices/
31. exp Ribavirin/
32. exp Antiviral Agents/
33. exp Social Distance/
34. exp Hypochlorous Acid/
35. exp Detergents/
36. exp Decontamination/
37. exp Disinfection/
38. exp Sterilization/
39. exp Hand Disinfection/
40. exp Soaps/
41. exp Filtration/
42. exp Inhalation Exposure/
43. prevention.mp.
44. control.mp.
45. communicable disease control.mp.
46. antisep*.mp.
47. isolat*.mp.
48. quarantin*.mp.
49. barrier nursing.mp.
50. hygiene.mp.
51. anti‐infective.mp.
52. disinfectant.mp.
53. gown.mp.
54. gloves.mp.
55. mask.mp.
56. goggles.mp.
57. eye protection.mp.
58. cohort nursing.mp.
59. ribavirin.mp.
60. palivizumab.mp.
61. antiviral.mp.
62. synagis.mp.
63. copegus.mp.
64. rebetol.mp.
65. virazole.mp.
66. airborne precautions.mp.
67. social distance.mp.
68. droplet precautions.mp.
69. respiratory hygiene.mp.
70. cough measures.mp.
71. personal protective devices.mp.
72. personal protective equipment.mp.
73. ppe.mp.
74. clean*.mp.
75. hypochlorite.mp.
76. detergent*.mp.
77. decontamin*.mp.
78. alcohol rub.mp.
79. handwashing.mp.
80. hand‐rub.mp.
81. particulate filter.mp.
82. 16 or 17 or 18 or 19 or 20 or 21 or 22 or 23 or 24 or 25 or 26 or 27 or 28 or 29 or 30 or 31 or 32 or 33 or 34 or 35 or 36 or 37 or 38 or 39 or 40 or 41 or 42 or 43 or 44 or 45 or 46 or 47 or 48 or 49 or 50 or 51 or 52 or 53 or 54 or 55 or 56 or 57 or 58 or 59 or 60 or 61 or 62 or 63 or 64 or 65 or 66 or 67 or 68 or 69 or 70 or 71 or 72 or 73 or 74 or 75 or 76 or 77 or 78 or 79 or 80 or 81
83. exp Respiratory Syncytial Virus, Human/
84. exp Bronchiolitis, Viral/
85. respiratory syncytial virus.mp.
86. rsv.mp.
87. bronchiolitis.mp.
88. 83 or 84 or 85 or 86 or 87
89. 15 and 88
90. 15 and 82 and 88
91. limit 89 to (English language and humans and year = ‘1860–2012’)
92. limit 90 to (English language and humans and year = ‘1860–2012’)

## References

[1] Nair H , Nokes DJ , Gessner BD *et al* Global burden of acute lower respiratory infections due to respiratory syncytial virus in young children: a systematic review and meta‐analysis. Lancet 2010; 375:1545–1555. 20399493 10.1016/S0140-6736(10)60206-1 PMC2864404

[2] Hall CB . Respiratory syncytial virus and parainfluenza virus. New Engl J Med 2001; 344:1917–1928. 11419430 10.1056/NEJM200106213442507

[3] Paes BA , Mitchell I , Banerji A *et al* A decade of respiratory syncytial virus epidemiology and prophylaxis: translating evidence into everyday clinical practice. Can Respir J 2011; 18:e10–e19. 21499597 10.1155/2011/493056 PMC3084427

[4] Falsey AR , Hennessey PA , Formica MA *et al* Respiratory syncytial virus infection in elderly and high‐risk adults. New Engl J Med 2005; 352:1749–1759. 15858184 10.1056/NEJMoa043951

[5] Murata Y , Falsey AR . Respiratory syncytial virus infection in adults. Antivir Ther 2007;12:659–670. 17944273

[6] Walsh EE . Respiratory syncytial virus infection in adults. Semin Respir Crit Care Med 2011; 32:423–432. 21858747 10.1055/s-0031-1283282

[7] Lee N , Lui GC , Wong KT *et al* High morbidity and mortality in adults hospitalized for respiratory syncytial virus infections. Clin Infect Dis 2013; 57:1069–1077. 23876395 10.1093/cid/cit471

[8] Volling C , Hassan K , Mazzulli T *et al* Respiratory syncytial virus infection‐associated hospitalization in adults: a retrospective cohort study. BMC Infect Dis 2014; 14:665. 25494918 10.1186/s12879-014-0665-2 PMC4269936

[9] Anak S , Atay D , Unuvar A *et al* Respiratory syncytial virus infection outbreak among pediatric patients with oncologic diseases and/or BMT. Pediatr Pulmonol 2010; 45:307–311. 20146398 10.1002/ppul.21184

[10] Kassis C , Champlin RE , Hachem RY *et al* Detection and control of a nosocomial respiratory syncytial virus outbreak in a stem cell transplantation unit: the role of Palivizumab. Biol Blood Marrow Transplant 2010; 16:1265–1271. 20304082 10.1016/j.bbmt.2010.03.011

[11] O'Connell K , Boo TW , Keady D *et al* Use of palivizumab and infection control measures to control an outbreak of respiratory syncytial virus in a neonatal intensive care unit confirmed by real‐time polymerase chain reaction. J Hosp Infect 2011; 77:338–342. 21330007 10.1016/j.jhin.2010.12.012

[12] Shachor‐Meyouhas Y , Zaidman I , Kra‐Oz Z *et al* Detection, control, and management of a respiratory syncytial virus outbreak in a pediatric hematology‐oncology department. J Pediatr Hematol Oncol 2013; 35:124–128. 23128340 10.1097/MPH.0b013e3182756edc

[13] Varga SM , Braciale TJ . The adaptive immune response to respiratory syncytial virus. Curr Top Microbiol Immunol 2013; 372:155–171. 24362689 10.1007/978-3-642-38919-1_8

[14] Broadbent L , Groves H , Shields M *et al* Respiratory syncytial virus, an ongoing medical dilemma: an expert commentary on respiratory syncytial virus prophylactic and therapeutic pharmaceuticals currently in clinical trials. Influenza Other Respir Viruses 2015; 9:169–178. 25847510 10.1111/irv.12313 PMC4474493

[15] Hall CB , Douglas RG Jr . Nosocomial respiratory syncytial viral infections. Should gowns and masks be used?. Am J Dis Child 1981; 135:512–515. 7234784 10.1001/archpedi.1981.02130300012006

[16] Isaacs D , Dickson H , O'Callaghan C *et al* Handwashing and cohorting in prevention of hospital acquired infections with respiratory syncytial virus. Arch Dis Child 1991; 66:227–231. 2001109 10.1136/adc.66.2.227 PMC1792812

[17] Leung AK , Kellner JD , Davies HD . Respiratory syncytial virus bronchiolitis. J Natl Med Assoc 2005; 97:1708–1713. 16396064 PMC2640754

[18] Madge P , Paton JY , McColl JH *et al* Prospective controlled study of four infection‐control procedures to prevent nosocomial infection with respiratory syncytial virus. Lancet 1992; 340:1079–1083. 1357462 10.1016/0140-6736(92)93088-5

[19] Dizdar EA , Aydemir C , Erdeve O *et al* Respiratory syncytial virus outbreak defined by rapid screening in a neonatal intensive care unit. J Hosp Infect 2010; 75:292–294. 20299133 10.1016/j.jhin.2010.01.013 PMC7132464

[20] Silva CDA , Dias L , Baltieri SR *et al* Respiratory syncytial virus outbreak in neonatal intensive care unit: impact of infection control measures plus palivizumab use. Antimicrob Resist Infect Control 2012; 1:16. 22958306 10.1186/2047-2994-1-16 PMC3441761

[21] Jefferson T , Del Mar CB , Dooley L *et al* Physical Interventions to Interrupt or Reduce the Spread of Respiratory Viruses. Cochrane Database of Systematic Reviews, John Wiley & Sons Ltd, 2011. 10.1002/14651858.CD006207.pub4 PMC6993921 21735402

[22] Beck CR , McKenzie B , Rajanaidu S *et al* Epidemiology of nosocomial RSV infection and clinical effectiveness of control measures to prevent transmission events: a systematic review: PROSPERO (University of York Centre for Reviews and Dissemination), 2013.

[23] Higgins JPT, Green SM (eds). Cochrane Handbook for Systematic Reviews of Interventions Version 5.1.0 [Updated March 2011]. The Cochrane Collaboration, 2011 Available at: www.cochrane-hhandbook.org (Accessed 10 January 2015).

[24] Moher D , Liberati A , Tetzlaff J *et al* Preferred reporting items for systematic reviews and meta‐analyses: the PRISMA statement. BMJ 2009; 339:b2535. 19622551 10.1136/bmj.b2535 PMC2714657

[25] Wells GA , Shea B , O'Connell D *et al* The Newcastle‐Ottawa Scale (NOS) for assessing the quality of nonrandomized studies in meta‐analysis. Secondary The Newcastle‐Ottawa Scale (NOS) for assessing the quality of nonrandomized studies in meta‐analysis. http://www.ohri.ca/programs/clinical_epidemiology/oxford.asp. (Accessed 5 July 2015).

[26] Centre for Reviews and Dissemination . Systematic Reviews. CRD's Guidance for Undertaking Reviews in Health Care. York: University of York, 2009.

[27] White MP , Mackie PL . Respiratory syncytial virus infection in special care nursery. Lancet 1990; 335:979. 1970055 10.1016/0140-6736(90)91047-e

[28] Alan S , Okulu E , Kilic A *et al* Palivizumab use during respiratory syncytial virus outbreak in the neonatal intensive care unit. J Hosp Infect 2012; 81:292–293. 22727129 10.1016/j.jhin.2012.05.011 PMC7134468

[29] Boedy R , Cox F , Kanto W *et al* Containment therapy and cost of a nosocomial respiratory syncytial virus RSV outbreak in a neonatal intensive care unit. Pediatr Res 1988;23:442A–42A.

[30] Halasa NB , Williams JV , Wilson GJ *et al* Medical and economic impact of a respiratory syncytial virus outbreak in a neonatal intensive care unit. Pediatr Infect Dis J 2005; 24:1040–1044. 16371862 10.1097/01.inf.0000190027.59795.ac

[31] Kilani RA . Respiratory syncytial virus (RSV) outbreak in the NICU: description of eight cases. J Trop Pediatr 2002; 48:118–122. 12022427 10.1093/tropej/48.2.118

[32] Kokstein Z , Basek P , Kalous P *et al* RSV outbreak in a neonatal intensive care unit. Perinatology 2001; 1:837–841.

[33] Kurz H , Herbich K , Janata O *et al* Experience with the use of palivizumab together with infection control measures to prevent respiratory syncytial virus outbreaks in neonatal intensive care units. J Hosp Infect 2008; 70:246–252. 18799241 10.1016/j.jhin.2008.07.013

[34] Meissner HC , Murray SA , Kiernan MA *et al* A simultaneous outbreak of respiratory syncytial virus and parainfluenza virus type 3 in a newborn nursery. J Pediatr 1984; 104:680–684. 6325655 10.1016/s0022-3476(84)80943-9

[35] Mintz L , Ballard RA , Sniderman SH *et al* Nosocomial respiratory syncytial virus infections in an intensive care nursery: rapid diagnosis by direct immunofluorescence. Pediatrics 1979; 64:149–153. 382079

[36] Visser A , Delport S , Venter M . Molecular epidemiological analysis of a nosocomial outbreak of respiratory syncytial virus associated pneumonia in a Kangaroo Mother Care unit in South Africa. J Med Virol 2008; 80:724–732. 18297695 10.1002/jmv.21128

[37] Krause HE , Saxtan DD , Mocegagonzalez H *et al* Role of hospital personnel in nosocomial respiratory syncytial virus (RSV) infections of children. Clin Res 1975; 23:A533.

[38] Salcedo S , Vinzo J , Calicó I *et al* Administration of palivizumab (monoclonal antibodies against respiratory syncytial virus) during a nosocomial outbreak in a neonatal unit [abstract #P241]. Prenatal Neonatal Med 2000; 5(Suppl 2):180.

[39] Abdallah A , Rowland KE , Schepetiuk SK *et al* An outbreak of respiratory syncytial virus infection in a bone marrow transplant unit: effect on engraftment and outcome of pneumonia without specific antiviral treatment. Bone Marrow Transplant 2003; 32:195–203. 12838285 10.1038/sj.bmt.1704116

[40] Harrington RD , Hooton TM , Hackman RC *et al* An outbreak of respiratory syncytial virus in a bone marrow transplant center. J Infect Dis 1992; 165:987–993. 1583345 10.1093/infdis/165.6.987

[41] Mangi MH , Aitken C , Newland AC . Outbreak of respiratory syncytial virus (RSV) infection in adult bone marrow transplant unit (BMT): role of ribavirin treatment and recommendations for prevention of RSV infection in high risk patients. Blood 1998; 92:334B–335B.

[42] Huang FL , Chen PY , Shi ZY *et al* An unusual respiratory syncytial virus nosocomial outbreak in an adult psychiatry ward. Jpn J Infect Dis 2009; 62:61–62. 19168963

[43] Mandal SK , Joglekar VM , Khan AS . An outbreak of respiratory syncytial virus infection in a continuing‐care geriatric ward. Age Ageing 1985; 14:184–186. 4013906 10.1093/ageing/14.3.184

[44] Agah R , Cherry JD , Garakian AJ *et al* Respiratory syncytial virus (RSV) infection rate in personnel caring for children with RSV infections. Routine isolation procedure vs routine procedure supplemented by use of masks and goggles. Am J Dis Child 1987; 141:695–697. 3578197 10.1001/archpedi.1987.04460060111049

[45] Gala CL , Hall CB , Schnabel KC *et al* The use of eye‐nose goggles to control nosocomial respiratory syncytial virus infection. JAMA 1986; 256:2706–2708. 3773177

[46] Krasinski K , LaCouture R , Holzman RS *et al* Screening for respiratory syncytial virus and assignment to a cohort at admission to reduce nosocomial transmission. J Pediatr 1990; 116:894–898. 2348292 10.1016/s0022-3476(05)80646-8

[47] Leclair JM , Freeman J , Sullivan BF *et al* Prevention of nosocomial respiratory syncytial virus infections through compliance with glove and gown isolation precautions. N Engl J Med 1987; 317:329–334. 3600729 10.1056/NEJM198708063170601

[48] Macartney KK , Gorelick MH , Manning ML *et al* Nosocomial respiratory syncytial virus infections: the cost‐effectiveness and cost‐benefit of infection control. Pediatrics 2000; 106:520–526. 10969097 10.1542/peds.106.3.520

[49] Simon A , Khurana K , Wilkesmann A *et al* Nosocomial respiratory syncytial virus infection: impact of prospective surveillance and targeted infection control. Int J Hyg Environ Health 2006; 209:317–324. 16697255 10.1016/j.ijheh.2006.02.003

[50] Murphy D , Todd JK , Chao RK *et al* The use of gowns and masks to control respiratory illness in pediatric hospital personnel. J Pediatr 1981; 99:746–750. 7299552 10.1016/S0022-3476(81)80401-5 PMC7173004

[51] Hall CB , Geiman JM , Douglas RG Jr *et al* Control of nosocomial respiratory syncytial viral infections. Pediatrics 1978; 62:728–732. 724317

[52] Langley JM , LeBlanc JC , Wang EEL *et al* Nosocomial respiratory syncytial virus infection in Canadian pediatric hospitals: a pediatric investigators collaborative network on infections in Canada study. Pediatrics 1997; 100:943–946. 9374561 10.1542/peds.100.6.943

[53] Hall CB . The shedding and spreading of respiratory syncytial virus. Pediatr Res 1977; 11:236–239. 846775

[54] Garcia R , Raad I , AbiSaid D *et al* Nosocomial respiratory syncytial virus infections: prevention and control in bone marrow transplant patients. Infect Control Hosp Epidemiol 1997; 18:412–416. 9181397 10.1086/647640

[55] Gardner PS , Court SD , Brocklebank JT *et al* Virus cross‐infection in paediatric wards. Br Med J 1973; 2:571–575. 4351623 10.1136/bmj.2.5866.571 PMC1592186

[56] Karanfil LV , Conlon M , Lykens K *et al* Reducing the rate of nosocomially transmitted respiratory syncytial virus. Am J Infect Control 1999; 27:91–96. 10196485 10.1016/s0196-6553(99)70087-8

[57] Katz BZ , Sullivan C . Respiratory syncytial virus prophylaxis in a tertiary care neonatal intensive care unit. Pediatr Infect Dis J 2009; 28:842–844. 19636282 10.1097/INF.0b013e3181a0ad01

[58] Lavergne V , Ghannoum M , Weiss K *et al* Successful prevention of respiratory syncytial virus nosocomial transmission following an enhanced seasonal infection control program. Bone Marrow Transplant 2011; 46:137–142. 20383207 10.1038/bmt.2010.67

[59] Raad I , Abbas J , Whimbey E . Infection control of nosocomial respiratory viral disease in the immunocompromised host. Am J Med 1997; 102:48–52. 10868143 10.1016/s0002-9343(97)00011-9

[60] Snydman DR , Greer C , Meissner HC *et al* Prevention of nosocomial transmission of respiratory syncytial virus in a newborn nursery. Infect Control Hosp Epidemiol 1988; 9:105–108. 3351266 10.1086/645804

[61] Page SD . Success of respiratory syncytial virus (RSV) isolation in preventing nosocomial RSV in a children's hospital. Am J Infect Control 2007; 35:E172–E173.

[62] Public Health England . Infection Control Precautions to Minimise Transmission of Acute Respiratory Tract Infections in Healthcare Settings. London: Public Health England, 2014; 1–14.

[63] Hall CB , Douglas RG Jr , Schnabel KC *et al* Infectivity of respiratory syncytial virus by various routes of inoculation. Infect Immun 1981; 33:779–783. 7287181 10.1128/iai.33.3.779-783.1981 PMC350778

[64] Feltes TF , Cabalka AK , Meissner HC *et al* Palivizumab prophylaxis reduces hospitalization due to respiratory syncytial virus in young children with hemodynamically significant congenital heart disease. J Pediatr 2003; 143:532–540. 14571236 10.1067/s0022-3476(03)00454-2

[65] IMpact‐RSV Study Group . Palivizumab, a humanized respiratory syncytial virus monoclonal antibody, reduces hospitalization from respiratory syncytial virus infection in high‐risk infants. The IMpact‐RSV Study Group. Pediatrics 1998; 102:531–537. 9738173

[66] Simpson S , Burls A . A Systematic Review of the Effectiveness and Cost‐Effectiveness of Palivizumab (Synagis) in the Prevention of Respiratory Syncytial Virus (RSV) Infection in Infants at High Risk of Infection. Birmingham: Department of Public Health & Epidemiology, University of Birmingham, 2003.

[67] Abadesso C , Almeida HI , Virella D *et al* Use of palivizumab to control an outbreak of syncytial respiratory virus in a neonatal intensive care unit. J Hosp Infect 2004; 58:38–41. 15350712 10.1016/j.jhin.2004.04.024

[68] Cox RA , Rao P , Brandon‐Cox C . The use of palivizumab monoclonal antibody to control an outbreak of respiratory syncytial virus infection in a special care baby unit. J Hosp Infect 2001; 48:186–192. 11439005 10.1053/jhin.2001.1002

[69] Heerens AT , Marshall DD , Bose CL . Nosocomial respiratory syncytial virus: a threat in the modern neonatal intensive care unit. J Perinatol 2002; 22:306–307. 12032794 10.1038/sj.jp.7210696

[70] Teale A , Deshpande S , Burls A . Palivizumab and the importance of cost effectiveness. BMJ (Online) 2009; 338:1474–1476.

[71] Gammon J , Morgan‐Samuel H , Gould D . A review of the evidence for suboptimal compliance of healthcare practitioners to standard/universal infection control precautions. J Clin Nurs 2008; 17:157–167. 17331098 10.1111/j.1365-2702.2006.01852.x

[72] Stone SP , Cooper BS , Kibbler CC *et al* The ORION statement: guidelines for transparent reporting of outbreak reports and intervention studies of nosocomial infection. Lancet Infect Dis 2007; 7:282–288. 17376385 10.1016/S1473-3099(07)70082-8

